# GhPLP2 Positively Regulates Cotton Resistance to Verticillium Wilt by Modulating Fatty Acid Accumulation and Jasmonic Acid Signaling Pathway

**DOI:** 10.3389/fpls.2021.749630

**Published:** 2021-11-02

**Authors:** Yutao Zhu, Xiaoqian Hu, Ping Wang, Linying Gao, Yakun Pei, Zhaoyue Ge, Xiaoyang Ge, Fuguang Li, Yuxia Hou

**Affiliations:** ^1^College of Science, China Agricultural University, Beijing, China; ^2^State Key Laboratory of Cotton Biology, Institute of Cotton Research, Chinese Academy of Agricultural Sciences, Anyang, China

**Keywords:** *Gossypium hirsutum*, patatin-like protein, *Verticillium dahliae*, fatty acid metabolism, jasmonic acid, disease resistance

## Abstract

Patatin-like proteins (PLPs) have non-specific lipid acyl hydrolysis (LAH) activity, which can hydrolyze membrane lipids into fatty acids and lysophospholipids. The vital role of PLPs in plant growth and abiotic stress has been well documented. However, the function of PLPs in plant defense responses against pathogens is still poorly understood. Here, we isolated and identified a novel cotton (*Gossypium hirsutum*) PLP gene *GhPLP2*. The expression of *GhPLP2* was induced upon treatment with *Verticillium dahliae*, the signaling molecules jasmonic acid (JA) and ethylene (ETH) in cotton plants. Subcellular localization revealed that GhPLP2 was localized to the plasma membrane. *GhPLP2*-silenced cotton plants were more susceptible to infection by *V. dahliae*, while the overexpression of *GhPLP2* in Arabidopsis enhanced its resistance to *V. dahliae*, which was apparent as mild symptoms, and a decrease in the disease index and fungal biomass. The hypersensitive response, deposition of callose, and H_2_O_2_ accumulation triggered by *V. dahliae* elicitor were reduced in *GhPLP2*-silenced cotton plants. The overexpression of *GhPLP2* in Arabidopsis resulted in the accumulation of linoleic acid (LA, 18:2) and α-linolenic acid (ALA, 18:3) and facilitated the biosynthesis of JA and JA-mediated defensive responses. *GhPLP2* silencing in cotton plants consistently reduced the accumulation of linoleic acid (LA, 18:2) and α-linolenic acid (ALA, 18:3) and suppressed the biosynthesis of JA and the defensive responses mediated by JA. These results indicate that *GhPLP2* is involved in the resistance of cotton to *V. dahliae* by maintaining fatty acid metabolism pools for JA biosynthesis and activating the JA signaling pathway.

## Introduction

Lipids in plants play a critical role in cell membrane components, the storage of sustainable energy, and signaling transduction in response to biotic and abiotic stress (Wang, 2004; Li et al., 2016; Lim et al., 2017). The accumulation of products of hydrolysis owing to the activity of lipid acyl hydrolase (LAH) in lipid metabolism affects pathogenesis and the mechanisms of resistance in plant-microbe interactions (Shah, 2005). As the key hydrolytic products of membrane lipids, fatty acids (FAs) participate in modulating the accumulation of endogenous nitric oxide and the biosynthesis of azelaic acid (AzA) which contribute to the development of systemic acquired resistance (SAR), production of reactive oxygen species (ROS), and subsequent defense responses in plants (Mandal et al., 2012; Yu et al., 2013; Wang et al., 2014). Oxidation and further conversions of polyunsaturated FAs by α-dioxygenase (α-DOX) and lipoxygenases (LOXs) generate various products that are collectively termed oxylipins (Savchenko et al., 2014; Cook et al., 2021). Jasmonates (JAs) constitute a crucial member of oxylipins, many of which are involved in plant defenses against pathogens, nematodes, and herbivores (Campos et al., 2014; Wasternack and Strnad, 2016, 2017). Moreover, oxylipins are related to the initiation of hypersensitive response (HR) in the interactions between plants and incompatible bacterial or fungal elicitors (Coll et al., 2011; García-Marcos et al., 2013; Sun et al., 2014).

Patatin proteins constitute a significant family of LAHs that possess non-specific LAH activity toward several membrane lipids; they were first identified and isolated as vacuolar storage proteins in potato tubers (Hendriks et al., 1991; Scherer et al., 2010). They generate free fatty acids and lysophospholipids by catalyzing the non-specific hydrolysis of membrane lipids, including phospholipids, glycolipids, sulfolipids, and mono- and diacylglycerols (Li and Wang, 2014). Various functions of patatin proteins have been demonstrated in responses to auxin, the production of cellulose, salt tolerance, drought response, and plant growth (Li et al., 2011; Yang et al., 2012; Dong et al., 2014; Li J. et al., 2020). More importantly, patatin and patatin-like proteins (PLPs) play an essential role in defense response to various pathogens. In Arabidopsis, patatin-related phospholipase I (AtPLAI) with the activity to hydrolyze acyls plays a role in maintaining the homeostatic pool of basal free fatty acids and basal jasmonic acid production and conferring resistance to infection with *Botrytis cinerea* (Yang et al., 2007). The Arabidopsis patatin-like protein 2 (AtPLP2), a pathogen-induced patatin-like LAH, provides fatty acids precursors for the biosynthesis of oxylipins and differentially affects the resistance to pathogens with distinct lifestyles (La Camera et al., 2009). Overexpression of Arabidopsis patatin-related phospholipase IIIα (AtpPLAIIIα) enhanced resistance to *turnip crinkle virus* (*TCV*), which was accompanied by a higher ratio of salicylic acid to jasmonic acid, and increased the level of expression of defense gene *PR1* (Jang et al., 2020). The silencing of pepper (*Capsicum annuum*) *CaPLP1* compromised the defense responses to avirulent *Xanthomonas campestris* pv. *vesicatoria* (*Xcv*) (Kim et al., 2014). Several PLPs are involved in releasing FAs from lipid membranes and serving as biosynthetic precursors of jasmonic acid synthesis in the grapevine *Vitis vinifera* cv. *Regent* (resistant cultivars) after infection with *Plasmopara viticola* (Laureano et al., 2018). Furthermore, three lipid acyl hydrolases (NtPATs) were rapidly induced in tobacco during its HR response to the *Tobacco mosaic virus* (*TMV*) (Dhondt et al., 2000).

Cotton (*Gossypium* spp.) is widely cultivated around the world because of its significant economic value to the textile industry (Gao et al., 2013). However, the yield and quality of cotton can be seriously affected by Verticillium wilt, caused by the soil-borne pathogen *Verticillium dahliae* Kleb (Gong et al., 2017; Dhar et al., 2020). *V. dahliae* is a hemibiotrophic fungus that undergoes biotrophic and necrotrophic phases during the process of infecting the host (Pantelides et al., 2010; Lo Presti et al., 2015; Zhang L. et al., 2017). It colonizes the plants through young roots, the sites of lateral root formation or puncture wounds to the xylem and leads to the browning of vasculature, defoliation, and wilting (Chen et al., 2016; Shaban et al., 2018). This pathogen can infect more than 200 species of dicotyledonous plants and is particularly difficult to control by fungicides because the fungus resides in the woody vascular tissues (Fradin and Thomma, 2006; Mo et al., 2016). Moreover, the resting structures microsclerotia of *V. dahliae* can survive in the soil for up to 14 years even without a host (Short et al., 2015). The cultivation of resistant cotton varieties has proven to be an effective strategy to control Verticillium wilt (Gayoso et al., 2010). The identification and isolation of candidate gene involved in defense against Verticillium wilt are essential to study the mechanism of resistance to this disease. Therefore, studying the function and mechanism of patatin proteins in the resistance of cotton to infection by *V. dahliae* may provide a new strategy for controlling Verticillium wilt.

Jasmonic acid (JA) is a vital lipid-derived phytohormone that confers the host resistance to biotrophic and hemibiotrophic pathogens (Ghorbel et al., 2021). Previous genetic evidence indicates that the JA signaling pathway is integral to the resistance of cotton to *V. dahliae* (Li et al., 2014; He et al., 2016; Dhar et al., 2020). The homeodomain transcription factor *HDTF1* identified from cotton plants infected with *V. dahliae* is a negative regulator of the JA signaling pathway. *HDTF1* silencing resulted in the activation of the JA signaling pathway and enhanced the resistance to *V. dahliae* and *B. cinerea* (Gao et al., 2016). The GhJAZ2 protein can interact with GhbHLH171 and inhibit its transcriptional activity, resulting in an impaired JA-mediated defense response against *V. dahliae* (He et al., 2018). The calcium-dependent protein kinase GhCPK33 from upland cotton (*Gossypium hirsutum*) phosphorylates GhOPR3, which leads to the enhanced instability of GhOPR3, which consequently limits the biosynthesis of JA and the subsequent resistance to *V. dahliae* (Hu et al., 2018). Silencing of the BEL1-Like transcription factor *GhBLH7-D06* enhanced the resistance of cotton to Verticillium wilt by activating the biosynthesis of lignin and the JA signaling pathway (Ma et al., 2020). The constitutive expression of the GSK3-like kinase BIN2 (Brassinosteroid insensitive 2) attenuated the resistance of plants to *V. dahliae* by regulating the endogenous content of JA and the expression of JA-responsive marker genes (Song et al., 2021).

In this study, we report the identification and characterization of PLP gene *GhPLP2* in cotton (*G. hirsutum*) that is induced by *V. dahliae*. The patterns of transcriptional expression of *GhPLP2* were studied in response to biotic and abiotic stress. Sequence analyses showed that GhPLP2 had conserved catalytic dyad residues, which are critical for the LAH activity of patatins. The recombinant GhPLP2 protein further confirmed its *in vitro* LAH activity. Reduced HR response during the *V. dahliae* elicitor infection was observed in *GhPLP2*-silenced plants, suggesting the role of *GhPLP2* in HR-like cell death signaling. The positive role of *GhPLP2* in regulating the resistance of cotton to *V. dahliae* was examined by virus-induced gene silencing (VIGS) technology in cotton plants and overexpression in Arabidopsis. Further experiments demonstrated that *GhPLP2* positively regulates the resistance of cotton to *V. dahliae* by mediating the metabolism of fatty acids and activation of the JA signaling pathway. Taken together, this research reveals the molecular mechanism of *GhPLP2* in the defense of cotton against *V. dahliae* and provides new insights to improve the resistance of cotton to Verticillium wilt.

## Materials and Methods

### Plant Growth and Culturing of *Verticillium dahliae*

Seeds of the upland cotton (*G. hirsutum*) resistant cultivar Zhongzhimian 2 (original strain no. GK44) were provided by the Cotton Research Institute, Chinese Academy of Agricultural Sciences (Anyang, Henan). The germinated seeds were cultured in nutrient soil and vermiculite (2:1, w/w) under a photoperiod of 16 h/8 h. After vernalization for 3 days at 4°C in Murashige-Skoog (MS) culture medium, both the wild-type (Columbia-0) and transgenic Arabidopsis plants were grown in a mixture of nutrient soil and vermiculite (1:1, w/w) under a controlled environment with 16 h light (25°C)/8-h dark (22°C). The aggressive defoliating isolate *Vd*991 of *V. dahliae* was cultured on potato dextrose agar (PDA) at 25°C for 7 days and then inoculated in Czapek liquid media. The experiments were performed using conidial suspensions of 1 × 10^7^ conidia/mL. Briefly, the cotton plants (2 weeks after VIGS) and Arabidopsis plants (4-week-old) were gently uprooted and the roots were dipped in conidial suspensions (1 × 10^7^ conidia/mL) for 5 min. Then they were replanted in a mixture of nutrient soil and vermiculite.

### Isolation of GhPLP2 cDNA

Total RNA was extracted using an RNA extraction kit (Biomed Gene Technology Co., Ltd., Wuhan, China), and the cDNA was synthesized using the manufacturer’s instructions with a FASTQuant cDNA RT Kit (Tiangen Biotech Co., Ltd., Beijing, China). The forward primer 5′-ACGCGTCGACATGGAAAAAAGTACTGGAAAC-3′ (*Sal*I, underlined restriction site) and the reverse primer 5′-ATAGACTAGTTGGGCCAAGCTTGTGCAA-3′ (*Spe*I, underlined restriction site) were designed according to the *GhPLP2* sequence (Gene ID: Gh_D02G0923) from cotton protein databases (*G. hirsutum*, NAU assembly) in the Cotton Functional Genomics Database^1^ to amplify the open reading frame (ORF) using the cDNA template. The PCR product was transferred into the pMD18-T vector according to the manufacturer’s instructions (TaKaRa, Dalian, China), and the positive clone was sequenced.

### Bioinformatic Analyses

The theoretical isoelectric point (pI) and molecular mass were calculated with ProtParam.^2^ All the sequences were retrieved from the website of the National Center for Biotechnology Information database,^3^ and the accession numbers of the patatin proteins are listed in Supplementary Table 1. Multiple amino acid sequence alignments were performed using Clustal Omega,^4^ and the multiple alignment file was shaded with BoxShade.^5^ A phylogenetic tree was constructed with the neighbor-joining method using MEGA 7 with bootstrap values from 1,000 replicates indicated at the nodes, and the motifs were annotated using MEME^6^ (Bailey et al., 2009; Kumar et al., 2016). The homology model of GhPLP2 was generated using SWISS-MODEL, and three-dimensional models were analyzed and visualized using EzMol, Version 1.20 (Bordoli et al., 2009; Reynolds et al., 2018). The crystal structure of SeMet Patatin (1oxw.1.B) was selected as a template to predict the theoretical model.

### Treatments With Methyl Jasmonate, Ethylene, PEG 6000, and *Verticillium dahliae*

For abiotic stresses and hormone treatments, 2-week-old cotton seedlings were gently uprooted and the roots were immersed in Hoagland solution that contained 100 μM methyl jasmonate (Sigma-Aldrich, St. Louis, MO, United States), 2 mM ethylene (Sigma-Aldrich), and 2.5% (w/v) PEG 6000, respectively (Champion et al., 2009; Pei et al., 2019). For the treatments with methyl jasmonate and ethylene, the whole plant was collected at 0, 0.5, 3, 6, 9, 12, 24, and 30 h for RNA extraction. For PEG 6000 treatment, the whole plant was collected at 0, 1, 3, 6, 12, 24, and 48 h for RNA extraction. For *V. dahliae* treatment, seedling roots were inoculated with conidial suspensions for 5 min and then transplanted into sterile soil. Control samples were treated with sterile water. The whole plant was collected at 0, 0.5, 12 h, 1, 3, 5, and 7 days for RNA extraction.

### Expression and Purification of the Recombinant GhPLP2 Protein

GhPLP2 was cloned into the 6 × His-Tagged protein expression vector pET-22b (+) (Novagen, Madison, WI, United States). The primers are listed in Supplementary Table 2. The recombinant vector was expressed in *Escherichia coli* BL21 (DE3). Single colonies were cultured at 37°C in LB broth that contained 100 μg/mL ampicillin. When the OD_600_ reached 0.6, the culture was induced with 0.4 mM IPTG for another 12 h at 22°C at 200 rpm. The protein was purified using a 6 × His-Tagged Protein Purification Kit (CWbio, Beijing, China) following the manufacturer’s instructions and detected using SDS-PAGE. The concentration of purified protein was determined using a Bradford Protein Assay Kit (TaKaRa, Dalian, China).

### GhPLP2 Enzyme Activity Assay Using the Substrates p-Nitrophenyl Palmitate and Phospholipids

The LAH activity of the GhPLP2 protein was assayed using the artificial substrate p-nitrophenyl palmitate (p-NPP) and phospholipids (Hong et al., 2008). Briefly, the reaction mixture was composed of 200 μL of Tris-HCl (pH 8.0), 10 mM CaCl_2_, 0.05% (v/v) Triton X-100, and 2 mM p-nitrophenyl palmitate to which 25 μg of purified protein was added and incubated at 37°C for 30 min. A volume of 700 μL of absolute ethanol was added and centrifuged for 2 min at 7,000 rpm. The absorbance of p-nitrophenol (NP) liberated from p-NPP was measured at 405 nm.

The substrates for phospholipase assay included 1,2-distearoyl-sn-glycerol-3-phosphoethanolamine (PE), 1,2-distearoyl-sn-glycerol-3-phosphocholine (PC), or L-α-phosphatidylglycerol (PG) (Aladdin, Shanghai, China). The reaction mixture was composed of 50 mM Tris-HCl (pH 8.0), 10 mM CaCl_2_, 0.05% (v/v) Triton X-100, 500 μg substrate, and 10 μg purified protein in a final volume of 600 μL. The reactions were performed at 37°C for 1 h and terminated by the addition of 500 μL chloroform: methanol (2:1, v/v). The fatty acids products released from the substrates were separated and analyzed as previously described (La Camera et al., 2005). Individual fatty acid methyl esters (FAME) were quantified using gas chromatography (GC) equipped with an Agilent column (Agilent Technologies, Santa Clara, CA, United States) (30 m by 0.25 mm, 0.25 μm film) and a flame ionization detector (FID).

### Subcellular Localization and Generation of Transgenic Plants

The PCR product with a *Sal*I/*Spe*I restriction site was inserted into a modified *pCAMBIA 1300* vector that harbored hygromycin phosphor-transferase (hptII) and green fluorescent protein (GFP) under the control of the CaMV 35S promoter (Supplementary Figure 1; Wang et al., 2011). By particle bombardment (PDS-1000; Bio-Rad, United States), the CaMV *35S:GhPLP2-GFP* and *35S:GFP* empty vector were transformed into onion epidermal cells. The transformed cells were incubated on the MS medium (with 20 g/L sucrose) in the dark for 18–24 h at 22°C.

For the generation of *GhPLP2*-transgenic plants, the construct was introduced into *Agrobacterium tumefaciens* strain GV3101 by the freeze-thaw method (Jyothishwaran et al., 2007), and Arabidopsis was transformed by the floral dip method (Clough and Bent, 1998). Transgenic Arabidopsis seeds were screened on MS plates (with 20 g/L sucrose) that contained 25 mg/mL hygromycin B and T3 homozygous lines that displayed 100% hygromycin resistance were used for additional experiments.

### Construction of Virus-Induced Gene Silencing Vectors and Agrobacterium-Mediated Virus-Induced Gene Silencing

The virus-induced gene silencing (VIGS) transient expression methods were previously described by Gao et al. (2011). The silenced fragments of *GhCLA1* (Cloroplastos alterados 1) and *GhPLP2* were amplified from cotton cDNA and inserted into the *TRV:00* vector to generate the *TRV:GhCLA1* and *TRV:GhPLP2* vectors. Then plasmids of *TRV:GhCLA1* and *TRV:GhPLP2* were transformed into *A. tumefaciens* strain GV3101 by heat shock (Dong et al., 2007). *TRV:GhCLA1* plants were used as positive controls. Two weeks after infiltration, when the *GhCLA1*-silenced plants showed clear signs of albinism in the leaves, the efficiency of *GhPLP2* was evaluated by quantitative real-time reverse transcriptase–PCR (qRT-PCR). *GhUBQ7* (DQ116441) was amplified as an internal control with the primers qUBQ7-F/R. All the primers are listed in Supplementary Table 2.

### *Verticillium dahliae* Inoculation and Disease Investigation

Two weeks after VIGS, when the true leaves of *GhCLA1*-silenced cotton plants showed clear signs of albinism, the plants were inoculated with *V. dahliae* (1 × 10^7^ conidia/mL) as previously described (Liu et al., 2018). The plant disease index (DI) was monitored using the following formula as described by Wang et al. (2020): DI = [Σ (n × the number of seedlings at level n)/(4 × the number of total seedlings)] × 100, n denotes disease level, cotton seedlings were divided into five levels based on their disease severity after inoculation with *V. dahliae* (level 0, 1, 2, 3, 4). For Arabidopsis plants, 4-week-old *GhPLP2*-transgenic and wild-type (WT) Arabidopsis plants were inoculated with *V. dahliae* spores as previously described (Gao et al., 2013). The controls were dipped in sterilized water. The disease index and classification of symptoms were determined as previously reported (Pei et al., 2019). Data were collected from three independent technical replicates (*n* ≥ 30).

The *V. dahliae* recovery assay was conducted and modified as described by Fradin et al. (2009), and the antibiotic in the PDA is kanamycin in this study. The stem section above the cotyledons was taken from cotton plants 3 weeks after inoculation with *V. dahliae*. Six slices were sterilized with 75% ethanol and 10% sodium hypochlorite solution and transferred onto PDA supplemented with kanamycin (50 mg/L) after the slices were washed three times with sterile water. The *V. dahliae* biomass was quantified as previously described (Ellendorff et al., 2009). Inoculated cotton (first internode) and Arabidopsis plants (whole plants) were harvested at 21 days post-inoculation (dpi) and 14 dpi, respectively. DNA was extracted from 100 mg of the fine powder. *V. dahliae* biomass was determined by qRT-PCR using fungus-specific *ITS1*-F (Liguori et al., 2007) and *V. dahliae*-specific *ST-Ve1*-R primers (Fradin et al., 2011). *GhUBQ7*-F/R (DQ116441) was used as a reference gene in the cotton plants, and *AtEF1*α-F/R (AT5G60390) was used as a reference gene in the Arabidopsis plants. All the primers are listed in Supplementary Table 2.

### Analysis of Hypersensitive Response in Cotton Induced by the PevD1 Elicitor From *Verticillium dahliae*

The primers were designed to obtain the *PevD1* fragment from *V. dahliae* (Supplementary Table 2), which was then cloned into the pET-28a (+) vector. The recombinant vector was expressed in *E. coli* BL21 (DE3), and the protein was induced with 0.1 mM IPTG for 10 h at 22°C while shaken at 200 rpm. The protein was purified using a 6 × His-Tagged Protein Purification Kit (CWbio, Beijing, China) following the manufacturer’s instructions and detected using SDS-PAGE. The concentration of purified protein was determined using a Bradford Protein Assay Kit (TaKaRa, Dalian, China). The treatment with PevD1 elicitor was performed as described by Li et al. (2019). The expression of HR marker genes *GhHSR203* and *GhHIN1* triggered by PevD1were detected in cotton leaves at 24 h after infection. Callose deposition was stained using 0.1% aniline blue as previously described (Wang et al., 2018). Images were observed using FLUOVIEW FV1000 on a confocal laser scanning microscope (Olympus, Tokyo, Japan). The level of H_2_O_2_ was measured by freshly made FOX reagent (Fe[NH_4_]_2_∙[SO_4_]_2_) (250 mM), xylenol orange (100 μM), sorbitol (100 μM), and H_2_SO_4_ (25 mM), and 1% ethanol as described by Pei et al. (2019).

### Fatty Acids Analysis and the Quantification of Hormones

Four-week-old Arabidopsis and 2-week *GhPLP2*-silenced cotton plants after VIGS were used to analyze the composition of fatty acids and quantify the hormones. The hormones were quantified as previously described (Li D. et al., 2020). Fresh samples were ground into fine powder in liquid nitrogen. A total of 100 mg powder was extracted with 1 mL of 80% aqueous methanol that contained 1% formic acid using an ultrasonic treatment at 4°C for 10 min and centrifuged at 12,000 rpm at 4°C for 10 min. The supernatant was transferred into a 2 mL centrifuge tube that contained 50 mg of primary secondary amine (CNW Technologies GmbH, Dusseldorf, Germany), dried with nitrogen, and added to 100 μL of 80% aqueous methanol that contained 1% formic acid. The samples were analyzed by an Agilent 6410B Triple Quadrupole HPLC-MS/MS (Agilent Technologies), which is equipped with an HPLC reverse phase C18 column (Athena C18-WP 2.1 × 50 mm, 3 μm). The flow rate was 0.3 mL/min. Methanol and ultrapure water were used as the mobile phases A and B, respectively. Isocratic elution was conducted for 5 min at a 65(A):35(B) ratio. MS was performed using the multiple reaction monitoring modes and negative electrospray ionization. All the parameters were performed as described by Li D. et al. (2020).

The fatty acid composition was analyzed by gas chromatography (GC) on Agilent 7890A (Agilent Technologies, Santa Clara, CA, United States) with a flame ionization detector (FID). Fatty acid methyl esterification was performed as described by Liu et al. (2019) with slight modification. The plant samples were dried at 90°C to a constant weight, and 50 mg of dry powder was weighed. Free fatty acids were methylated in 1 mL 0.5 mol/L KOH-methanol at 60°C for 2 h. Next, 1 mL of hexane that contained 0.01% butylated hydroxytoluene (BHT) and 0.1 mg methyl non-adecanoate was added. After shaking and standing for stratification, the supernatant that contained the FAMEs was separated. The GC parameters were as follows: 180°C for 10 min followed by a ramp to 190°C at 1°C/min, a hold at 190°C for 3 min, and then heating up to 220°C with a gradient at 4°C/min. The final temperature was maintained for 3 min.

### Plant Drought Treatment

Arabidopsis drought treatment was carried out as previously described (Wang et al., 2018). Briefly, water was withheld from 3-week-old Arabidopsis plants for 16 days, followed by watering for 2 days from the bottom. All the Arabidopsis plants were grown in a controlled environment with 16 h light (25°C)/8-h dark (22°C) and a relative humidity at 80%.

### Gene Expression Analysis by Quantitative Real-Time PCR

Total RNA was obtained from the plant samples with an RNA extraction kit with DNase I (Biomed Gene Technology, Co., Ltd., Beijing, China). The cDNA was synthesized using a PrimeScript^TM^ RT reagent Kit with gDNA Eraser (Perfect Real Time) (TaKaRa Bio, Dalian, China). qRT-PCR was performed using SYBR Premix Ex Taq (Tli RNaseH Plus) (TaKaRa, Dalian, China) on an ABI 7500 thermocycler (Applied Biosystems, Foster City, CA, United States). *AtEF1a* (AT5G60390) and *GhUBQ7* (DQ116441) were utilized as internal standard genes in Arabidopsis and cotton, respectively. The relative gene expression was calculated using the 2^−ΔΔCt^ method (Livak and Schmittgen, 2001). The melting curves for each gene studied (with a no template control) are provided in Supplementary Figures 2, 3. The primer sequences are listed in Supplementary Table 2.

### Data Analysis

The data were obtained from three independent technical or biological replicates per treatment and presented as the mean ± standard error. The statistical analyses were performed by Student’s *t*-test. Asterisks indicates a significant difference between the groups (*^∗^P* < 0.05, *^∗∗^P* < 0.01).

## Results

### Identification and Sequence Analysis of GhPLP2

**FIGURE 1:**
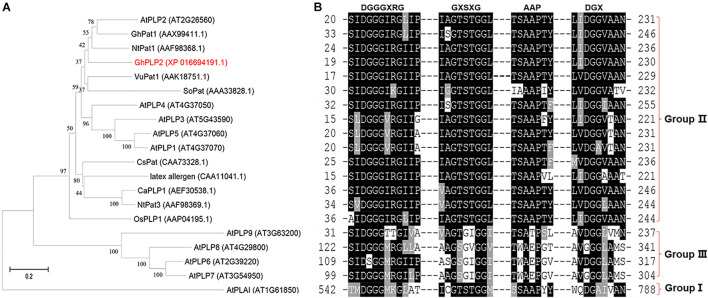
Sequence analyses of GhPLP2 and other patatin-like proteins. **(A)** Phylogenetic analysis of GhPLP2 and other plant patatin-like proteins. At1g61850 (AtPLAI) was used as the outgroup. The numbers on the tree represent bootstrap scores. **(B)** Alignment of the conserved motifs of patatin-like proteins. Esterase box GxSxG and residues DGX constitute the S-D catalytic dyad. Anion binding element: DGGGxxG and proline motif: APP.

From the transcriptome analysis of the cotton cultivar Zhongzhimian 2 that is resistant to infection by *V. dahliae Vd*991, the PLP gene *GhPLP2* was highly induced when the plants were infected with *V. dahliae*. We isolated the full-length clone of *GhPLP2* that consists of a 68 bp 5′untranslated region (5′ UTR), 138 bp 3′ UTR, and 1,218 bp open reading frame (ORF) that encoded a 405 amino acid protein with a theoretical pI of 8.21 and a molecular weight of 44.44 kDa. The GhPLP2 protein contains the conserved serine (S) hydrolase motif GXSXG at residues 63–67 and a conserved aspartic acid (D) residue at 214 within the patatin domain (residues 21–221) (Supplementary Figure 4). The catalytic dyad of S and D residues is critical for the LAH activity of patatin (Rydel et al., 2003; Rietz et al., 2010). Using SeMet patatin, an isozyme of patatin 17 from *Solanum cardiophyllum* as the template, the homology modeling results indicate that the Ser-Asp catalytic dyad is essential for the LAH activity of GhPLP2 (Supplementary Figure 5). GhPLP2 lacks the transmembrane domain or signal peptide. To study the possible role of GhPLP2 protein in cotton plants, a phylogenetic analysis was generated with homologous patatin proteins with precise functions that had been previously reported from other species. The phylogenetic analysis showed that plant patatins were divided into three groups, and GhPLP2 is located in group II that contains NtPat1 (Cacas et al., 2005), GhPat1 (Cacas et al., 2009), CaPLP1 (Kim et al., 2014), and AtPLP2 (La Camera et al., 2009; Figure 1A). The patatin homologs were divided into three groups based on the conserved motif alignments, which are consistent with the phylogenetic analysis result (Figure 1B). Group II members have the canonical S-D dyad esterase motif constituted by GxSxG and DGG/A in patatin catalytic centers, a conserved anion binding element DGGGxxG and the proline motif APP. GhPLP2 is a patatin group II member with the conserved catalytic dyad residues.

### Lipid Acyl Hydrolase Activity Assay of GhPLP2

**FIGURE 2:**
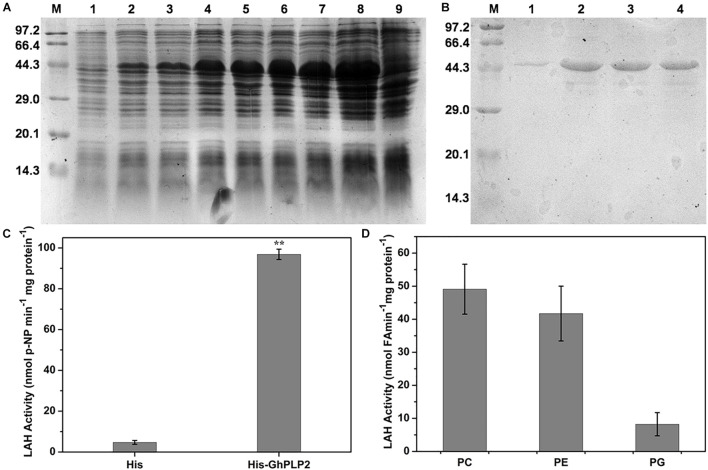
Induction and purification of the His-GhPLP2 protein and the LAH activity assay. **(A)** Proteins from induced bacteria were analyzed by SDS-PAGE. M: molecular mass of markers (kDa); 1: uninduced crude bacterial proteins; 2–8: IPTG-induced crude bacterial proteins at 22°C with different time points (1, 2, 4, 6, 12, 16, and 24 h); 9: pET-22b empty vector was induced at 24 h. **(B)** GhPLP2 fusion proteins purified on a Ni-column. 1–4: a series of eluted proteins. **(C)** p-nitrophenol (NP) was liberated from p-nitrophenyl palmitate by the LAH activity of GhPLP2 protein. Asterisks indicate a significant difference compared with the empty vector control (**P < 0.01, Student’s *t*-test). **(D)** Fatty acids were released by the activity of LAH from the GhPLP2 protein when PC, PE, and PG were used as substrates. Data were obtained from three independent biological replicates and presented as the mean ± standard error.

The LAH activity of patatin proteins can hydrolyze membrane lipids into free fatty acids and lysophosphatidic acid, which lead to a series of signaling pathways involved in growth development and defense responses (Ryu, 2004). To understand the role of GhPLP2 in more detail, the LAH activity of the recombinant GhPLP2 protein was studied. The recombinant protein GhPLP2 was produced and purified from *E. coli* BL21 (DE3). The GhPLP2 protein was induced with 0.4 mM IPTG at 22°C and as early as 1 h after induction (Figure 2A). The GhPLP2 fusion protein was purified using Ni columns, and the elution fractions were confirmed by SDS-PAGE (Figure 2B). When p-NPP was used as the substrate, the His-GhPLP2 protein exhibited high LAH activity (97 nmol min^–1^ mg^–1^), and the vector control was totally deficient in enzyme activity (Figure 2C). In addition, when the phospholipids PC, PE, and PG were used as substrates, the release of free fatty acids was quantified. The purified GhPLP2 protein released fatty acids from these phospholipids and exhibited variant enzyme activities in different phospholipid substrates (Figure 2D). The results indicate that GhPLP2 may function as a LAH to release free fatty acids in plants.

### Subcellular Localization of the GhPLP2 Protein

**FIGURE 3:**
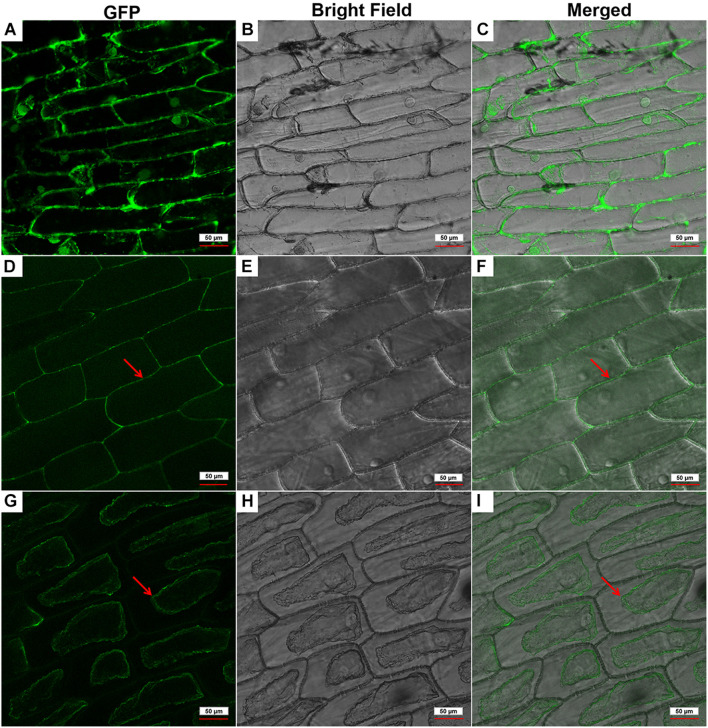
Subcellular localization of the GhPLP2-GFP fusion protein in onion epidermal cells. **(A–C)** *35S:GFP* empty vector was used as a control. **(D–F)** Red arrows indicated the localization of the GhPLP2-GFP fusion protein. **(G–I)** Red arrows indicated the localization of the GhPLP2-GFP fusion protein after plasmolysis. Scale bar represents 50 μm.

To determine the subcellular localization of GhPLP2 protein, the GFP signals of GhPLP2-GFP fusion protein and 35S:GFP empty vector were monitored by Nikon A1R SI Confocal (Nikon, Japan). The GFP signal of 35S:GFP empty vector was diffused throughout the cell (Figures 3A–C), whereas the GhPLP2-GFP fusion protein was found to accumulate exclusively in the cell wall or plasma membrane (Figures 3D–F). To further clarify the location of GhPLP2, the onion epidermal cells were treated with 0.8 M mannitol for 10 min. After plasmolysis, the GFP signals of GhPLP2-GFP fusion protein was found in the plasma membrane (Figures 3G–I). These results revealed that GhPLP2 was localized to the plasma membrane.

### Induction of GhPLP2 by Various Stresses

**FIGURE 4:**
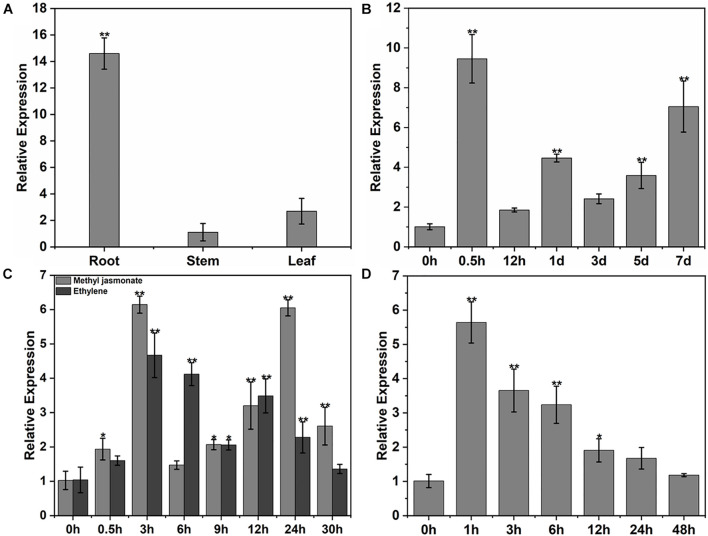
Patterns of expression of *GhPLP2* in cotton subjected to different types of stress. **(A)** The patterns of expression of *GhPLP2* in different tissues. **(B)** Expression of *GhPLP2* after inoculation with *Verticillium dahliae*. **(C)** Expression of *GhPLP2* after treatment with 100 μM methyl jasmonate and 2 mM ethylene. **(D)** Expression of *GhPLP2* after treatment with 2.5% PEG 6000 (w/v). Data were obtained from three independent biological replicates and presented as the mean ± standard error. Asterisks indicate a significant difference compared with the control. (*P < 0.05, **P < 0.01, Student’s *t*-test).

Expression of the *GhPLP2* gene in cotton was examined by qRT-PCR. *GhPLP2* was the most abundant in cotton roots (Figure 4A), which are the first physical barrier in cotton plants against infection by *V. dahliae*. The change in expression of *GhPLP2* in response to various stresses was investigated. The expression of *GhPLP2* was significantly increased by 9.4- and 7.1-fold at 0.5 h and 7 days after inoculation with *V. dahliae*, respectively (Figure 4B). We investigated the expression of *GhPLP2* after treatment with the defense-related signaling molecules methyl jasmonate and ethylene in more detail. The expression of *GhPLP2* was significantly higher at 3 h by 6.2-fold, and another 6.0-fold peak appeared at 24 h after treatment with JA (Figure 4C). In contrast, ethylene increased the expression of *GhPLP2* at 0.5 h, with a maximum level of 4.7-fold observed at 3 h (Figure 4C). The treatment with 2.5% PEG 6000 (w/v) markedly increased the gene expression at 1 and 3 h with 5.6- and 3.7-fold, respectively (Figure 4D).

### Enhanced Disease Susceptibility of GhPLP2-Silenced Cotton Plants to *Verticillium dahliae*

**FIGURE 5:**
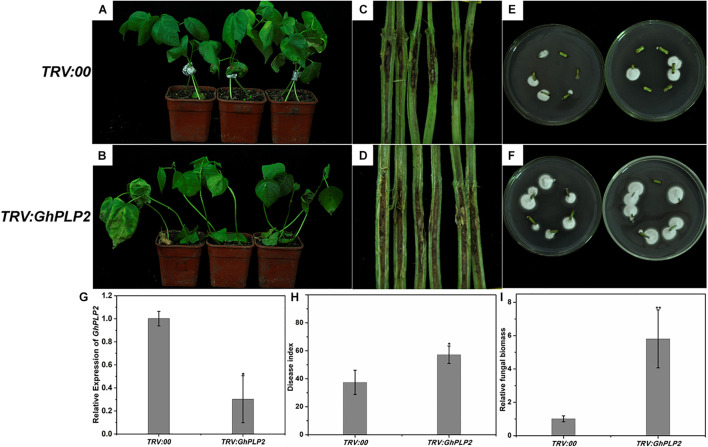
Attenuated resistance of *GhPLP2*-silenced cotton plants to *Verticillium dahliae*. Disease phenotypes of the control **(A)** and *GhPLP2*-silenced cotton plants **(B)** inoculated with *V. dahliae* at 14 days. Vascular browning in the stems of control **(C)** and *GhPLP2*-silenced cotton plants **(D)** infected by *V. dahliae* at 21 days. The fungal renewal cultivation of control **(E)** and *GhPLP2*-silenced cotton plants **(F)** infected by *V. dahliae* at 21 days. **(G)** The VIGS efficiency of *GhPLP2* was evaluated by qRT-PCR after 2 weeks. Data were obtained from three independent biological replicates. Asterisks indicate a significant difference compared with the *TRV:00*. **(H)** The disease index of control and *GhPLP2*-silenced cotton plants inoculated with *V. dahliae* at 14 days. Data were obtained from three independent technical replicates. Asterisks indicate a significant difference compared with *TRV:00*. **(I)** Relative fungal biomass of the control and *GhPLP2*-silenced cotton plants infected by *V. dahliae* at 21 days. Data represent the means ± standard error of three independent biological replicates. Asterisks indicate a significant difference compared with *TRV:00*. (*P < 0.05, **P < 0.01, Student’s *t*-test).

The *GhPLP2* gene was silenced by VIGS in cotton plants to clarify its function by Gao et al. (2013). The cotton gene *GhCLA1*, which is involved in chloroplast development, was used as a positive control (Gao et al., 2011). To assess its gene silencing efficiency, the expression of *GhPLP2* was monitored by semi-quantitative PCR and qRT-PCR in *TRV:00* and *TRV:GhPLP2* cotton true leaves after 2 weeks of VIGS (Figure 5G and Supplementary Figure 6). The results indicated that the expression of *GhPLP2* was effectively reduced in *GhPLP2*-silenced cotton plants.

To analyze the involvement of *GhPLP2* in the interaction between cotton and *V. dahliae*, cotton plants were inoculated with this pathogen. After inoculation, typical disease symptoms appeared at 10 days in *GhPLP2*-silenced cotton. At 14 days, leaf chlorosis and necrosis were more severe in the *GhPLP2*-silenced plants (Figures 5A,B). Dark and necrotic vascular bundles of the dissected stems were more apparent in *GhPLP2*-silenced plants at 21 days (Figures 5C,D). The fungal renewal cultivation showed that more fungi were recovered from *GhPLP2*-silenced plants compared with the control (Figures 5E,F). The plant disease index of the silenced plants was higher than that of the control plants (Figure 5H). In addition, the fungal biomass of the stems from the *GhPLP2*-silenced plants were higher than that in the control plants determined by qRT-PCR analysis (Figure 5I). These results suggest that the silencing of *GhPLP2* attenuates the resistance of cotton to *V. dahliae*.

### Silencing of GhPLP2 Compromises the Hypersensitive Response Symptoms Triggered by *Verticillium dahliae* Elicitor PevD1

**FIGURE 6:**
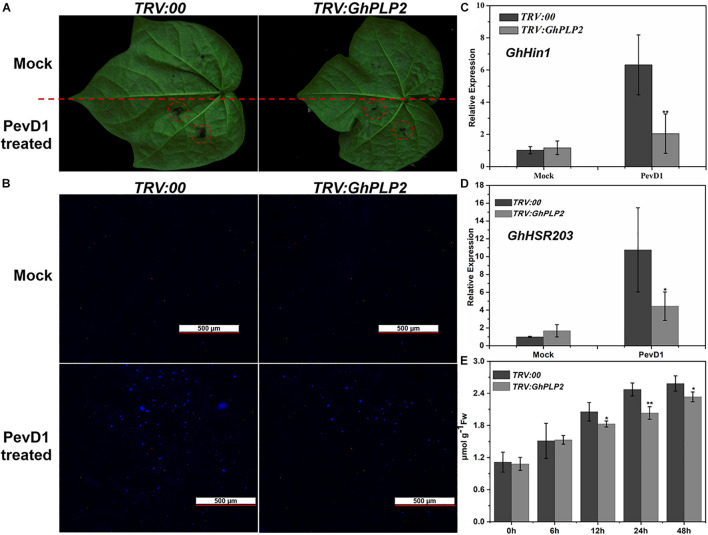
*GhPLP2*-mediated HR triggered by the *Verticillium dahliae* elicitor PevD1. **(A)** HR phenotypes developed on the leaves after injection with the PevD1 elicitor. **(B)** Callose deposition on the infected leaves at 24 h after injection. Scale bar represents 500 μm. **(C,D)** The expression of HR marker genes *GhHIN1* and *GhHSR203*. **(E)** Detection of H_2_O_2_ in the leaves during injection with PevD1. Data were obtained from three independent biological replicates. Asterisks indicate a significant difference, as determined by Student’s *t*-test. (*P < 0.05, **P < 0.01).

The HR is an efficient and immediate resistance reaction during the attack of pathogens, which rapidly causes cell death around the attempted infection site that restricts the spread of pathogens (Vailleau et al., 2002). During the HR, plant immune responses, including the accumulation of H_2_O_2_, deposition of callose, and the expression of HR-related marker genes were rapidly induced (Hamada et al., 2005; Simon et al., 2010; Rossi et al., 2011). PLPs have been shown to participate in HR in response to infection by the Gram-negative bacterial pathogen *Xanthomonas campestris* pv. *vesicatoria* in pepper plants and the response to *Tobacco mosaic virus* (*TMV*) in tobacco plants (Dhondt et al., 2000; Choi and Hwang, 2011; Kim et al., 2014). Therefore, *GhPLP2* may regulate the resistance to *V. dahliae* by mediating an HR induced by *V. dahliae* elicitors. To test our hypothesis, we expressed and purified PevD1 from *V. dahliae* (Supplementary Figure 7), which triggered HR and resistance responses in cotton, Arabidopsis, and tobacco (Wang et al., 2012; Bu et al., 2014; Liu et al., 2016). The HR-like cell death was initiated at 24 h after injection with PevD1, while it was significantly reduced in *GhPLP2*-silenced leaves (Figure 6A). Moreover, PevD1 triggered the deposition of callose, and the expression of HR marker genes *GhHIN1* and *GhHSR203* were compromised in the *GhPLP2*-silenced leaves at 24 h after the infiltration (Figures 6B–D). In addition, the silencing of *GhPLP2* significantly compromised the production of H_2_O_2_ induced by PevD1 (Figure 6E).

### Overexpression of GhPLP2 in Arabidopsis Confers Enhanced Resistance to *Verticillium dahliae*

To further evaluate the role of *GhPLP2* in response to *V. dahliae*, we examined the resistance of wild-type (WT) and transgenic Arabidopsis seedlings to *V. dahliae* infection. Three homozygous transgenic (T3 generation) lines with the highest levels of expression of *GhPLP2* (Lines 1, 4, and 8) were selected for additional experiments (Supplementary Figure 8).

**FIGURE 7:**
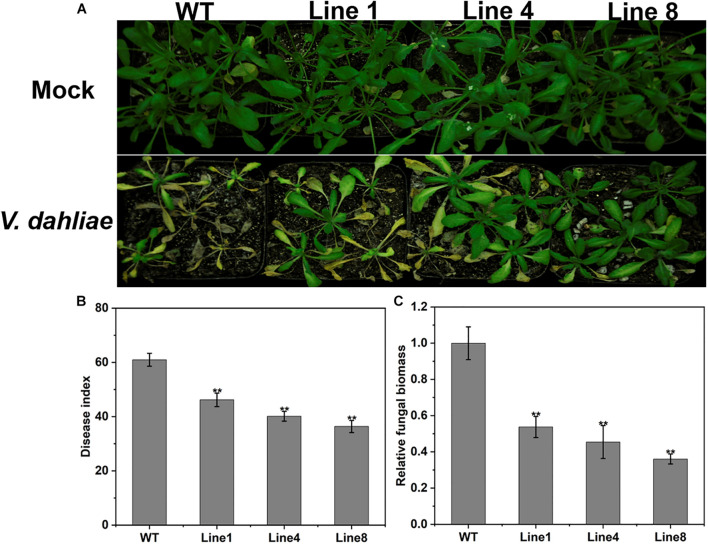
Increased resistance to *Verticillium dahliae* in *GhPLP2*-overexpressed Arabidopsis. **(A)** Arabidopsis plants were inoculated with sterile water and *V. dahliae* by root dipping, respectively. Images were taken at 14 dpi. **(B)** Disease index at 14 dpi. Data represent the means ± standard error of three independent technical replicates. Asterisks indicate a significant difference compared with the WT. (**P < 0.01, Student’s *t*-test). **(C)** The fungal biomass of Arabidopsis plants at 14 dpi. Data represent the means ± standard error of three independent biological replicates. Asterisks indicate a significant difference compared with the WT. (**P < 0.01, Student’s *t*-test).

Four-week-old transgenic and WT plants were infected with *V. dahliae* spores by the root dipping method (Pei et al., 2019). After inoculation, the WT plants showed more serious wilting, yellowish coloring and necrosis compared with the transgenic plants at 14 dpi (Figure 7A); this was consistent with the disease index investigation (Figure 7B). The fungal biomass of the WT plants was remarkably high compared with that on the transgenic plants as determined by qRT-PCR analysis (Figure 7C). These results indicate that the overexpression of *GhPLP2* in Arabidopsis plants confers enhanced resistance to *V. dahliae*.

### GhPLP2 Involved in the Accumulation of Fatty Acids and Jasmonic Acid Signaling Pathway

**FIGURE 8:**
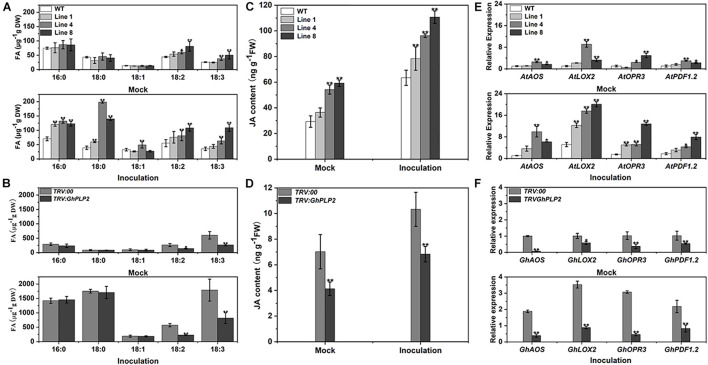
The composition of fatty acids, accumulation with JA, and gene expression were determined in Arabidopsis and cotton plants 3 days after inoculation with water (mock) and *Verticillium dahliae*. **(A)** Analysis of the fatty acids composition in Arabidopsis plants 3 days after inoculation with water (mock) and *V. dahliae*. **(B)** Analysis of the fatty acids composition in cotton plants 3 days after inoculation with water (mock) and *V. dahliae.* **(C)** Accumulation of JA in Arabidopsis plants 3 days after inoculation with water (mock) and *V. dahliae*. **(D)** Accumulation of JA in cotton plants 3 days after inoculation with water (mock) and *V. dahliae*. **(E)** The relative level of expression of the genes associated with the JA signaling pathway in WT and *GhPLP2*-overexpressed Arabidopsis plants 3 days after inoculation with water (mock) and *V. dahliae*. *AtEF1*α (AT5G60390) was used as the standard internal gene. **(F)** The relative expression of genes associated with the JA signaling pathway in *TRV:00* and *GhPLP2*-silenced cotton plants 3 days after inoculation with water (mock) and *V. dahliae*. *GhUBQ7* (DQ116441) was used as the standard internal gene. Data represent the means ± standard error of three independent biological repeats. Asterisks indicate a significant difference compared with WT or *TRV:00*, respectively. (*P < 0.05, **P < 0.01, Student’s *t*-test).

Free fatty acids (FFAs) are among the hydrolyzates of membrane lipids owing to PLPs with LAH activity (Rivas and Heitz, 2014). The endogenous LAH activity of crude proteins from different genotypes plants was assayed using p-NPP as the substrate (Lin et al., 2011). The LAH activity of *GhPLP2*-overexpressed Arabidopsis lines was higher than that of the WT plants, whereas the LAH activity decreased in *TRV:GhPLP2* cotton plants compared with the *TRV:00* plants (Supplementary Figure 9). To further assess the metabolic differences between different genotypes of plants, the composition of fatty acids was examined in Arabidopsis and cotton plants 3 days after the inoculation of *V. dahliae* and water (mock) (Figure 8). As shown in Figure 8A, the analyses of the composition of fatty acids indicated that the levels of linoleic acid (LA, 18:2) and α-linolenic acid (ALA, 18:3) were higher in *GhPLP2*-overexpressed Arabidopsis plants than in the WT plants under treatment with water. After inoculation with *V. dahliae*, the content of different types of fatty acids increased in both genotypes of Arabidopsis plants, and the increase in *GhPLP2*-overexpressed Arabidopsis plants was more apparent compared with the WT (Figure 8A). Moreover, *GhPLP2*-silenced cotton plants exhibited a significant reduction in their contents of LA and ALA regardless of the treatment with *V. dahliae* (Figure 8B). In addition, the content of palmitic acid (16:0) and stearic acid (18:0) also increased significantly after the inoculation of cotton plants with *V. dahliae*.

It has been reported that the oxidation and further conversion of C18 polyunsaturated FAs with lipoxygenases (LOXs) may generate the production of JA (Walley et al., 2013; Sun et al., 2014; Fu et al., 2015). We then investigated the JA contents in *GhPLP2*-overexpressed Arabidopsis and *GhPLP2*-silenced cotton plants. The contents of JA in *GhPLP2*-overexpressed Arabidopsis lines 4 and 8 were 84.97 and 101.99% higher than that of the WT, respectively, when they had not been inoculated with *V. dahliae*. After inoculation with *V. dahliae*, the JA contents of *GhPLP2*-overexpressed Arabidopsis lines 4 and 8 were 51.63 and 74.42% higher than that of the WT, respectively (Figure 8C). The levels of JA in the *TRV:GhPLP2* cotton plants were 41.21 and 33.83% lower than that of the *TRV:00* cotton plants under mock and *V. dahliae* inoculations, respectively (Figure 8D).

We also conducted qRT-PCR to detect the expression of a series of the JA-synthesis and -signaling genes from Arabidopsis and cotton plants, namely *AOS*, *LOX2*, *OPR3*, and *PDF1.2*. All of these genes were highly induced by infection with *V. dahliae* in both Arabidopsis and cotton at 3 dpi (Figures 8E,F). Consistent with the contents of JA, we found that the expression of these genes was significantly upregulated in *GhPLP2*-overexpressed Arabidopsis plants compared with the WT and downregulated in *TRV:GhPLP2* cotton plants relative to the *TRV:00* plants in both treatments (Figures 8E,F). Taken together, the results showed that *GhPLP2* contributes to the accumulation of fatty acids and is involved in the JA signaling pathway in both Arabidopsis and cotton plants.

### GhPLP2 Overexpression Has No Effect on Arabidopsis Tolerance to Drought

Based on our observation that PEG 6000 induced the expression of *GhPLP2*, we investigated the drought tolerance of *GhPLP2*-overexpressed Arabidopsis plants. However, the *GhPLP2*-overexpressed Arabidopsis did not show the distinct phenotype of drought sensitivity compared with the WT plants (Supplementary Figure 10).

## Discussion

Patatin-like proteins (PLPs) are a significant family of lipases with LAH activity and play an essential role in lipid metabolism during the immunity that arises from plant defenses (Rivas and Heitz, 2014). In this study, we identified the novel PLP gene *GhPLP2* from cotton and demonstrated its role in fatty acid metabolism and the JA signaling pathway that confers resistance to *V. dahliae* in cotton.

PLPs have a conserved S-D catalytic dyad that is composed of serine of esterase motifs (GxSxG) and a central aspartic acid, which is essential for the LAH activity (Rydel et al., 2003). These residues were also identified in human Ca^2+^-independent phospholipase A2 (iPLA2s) and many microbial proteins (Burke and Dennis, 2009; Mansfeld, 2009; Heller et al., 2018). Similar to other patatin proteins in plants, GhPLP2 has the canonical esterase motif and the conserved S-D catalytic dyad for its LAH activity (Figure 1B). GhPLP2 is localized in the plasma membrane (Figure 3), which was consistent with the localization of several PLPs (Holk et al., 2002; Kim et al., 2014). The localization on the plasma membrane suggests that GhPLP2 may be involved in plant physiology and defense responses through the activation of lipid signaling by the hydrolysis of membrane lipids. The *in vitro* enzyme activity assay confirmed that GhPLP2 has LAH activity (Figures 2C,D) as it has been reported for other plant PLPs (Holk et al., 2002; Li and Wang, 2014). The *GhPLP2*-overexpressed Arabidopsis plants and *GhPLP2*-silenced cotton plants were utilized to investigate the *in vivo* function of *GhPLP2* on plant fatty acid metabolism. The overexpression of *GhPLP2* in Arabidopsis enhanced the levels of linoleic acid (LA, 18:2) and α-linolenic acid (ALA, 18:3) relative to the WT (Figure 8A) not only after inoculation with *V. dahliae* but also after the water treatment. In contrast, the knockdown of *GhPLP2* in cotton plants reduced the contents of both types of FAs (Figure 8B). The functional LAH activity of GhPLP2 indicates its ability to release fatty acids from membrane lipids.

Many studies have suggested that PLPs with soluble LAH activity are necessary for the response of plants to various environmental stresses, such as drought, wounding, or pathogen infections (Matos and Pham-Thi, 2009; Rivas and Heitz, 2014). PLP proteins in tobacco, Arabidopsis, pepper, and grape show distinct resistance mechanisms in different host-pathogen interactions (Cacas et al., 2005; Yang et al., 2007; La Camera et al., 2009; Kim et al., 2014; Laureano et al., 2018). The strong upregulation of the *GhPLP2* gene upon infection by *V. dahliae* (Figure 4B) suggests its crucial role in the interaction of cotton plants with *V. dahliae*. The silencing of *GhPLP2* in cotton plants enhanced their susceptibility to infection by *V. dahliae* (Figure 5), and the overexpression of *GhPLP2* in Arabidopsis plants visibly enhanced their resistance to disease through the appearance of merely mild symptoms of disease (Figure 7), demonstrating that *GhPLP2* played a positive role in the plant defense to infection with *V. dahliae*.

Previous studies have shown that the JA biosynthetic and signaling pathway are important for plant resistance against *V. dahliae* (Li et al., 2014; He et al., 2016; Dhar et al., 2020). *GhPLP2* was highly induced after JA treatment (Figure 4C), suggesting that *GhPLP2* is likely to involve JA-dependent defense pathway. Several studies have shown that PLPs mediate plant immune responses through the regulation of FA metabolism and the JA signaling pathway. In tobacco, PLP was rapidly induced before the accumulation of JA in response to infection by the *Tobacco mosaic virus* (*TMV*) (Dhondt et al., 2002). AtPLAI targets the chloroplasts and plays a critical role in maintaining the homeostatic pool of free FA and basal JA, thus, increasing resistance to *B. cinerea* (Yang et al., 2007). The branch of the JA family (OPDA, JA, and dinor-OPDA) are fed by the primary cytoplasmic localization protein AtPLP2 (La Camera et al., 2009). The overexpression of the PLP gene *OSAG78* in Arabidopsis increased the amounts of linoleic acid and linolenic acid and induced the expression levels of the JA-related defense genes *PDF 1.2* and *PR4* (Lin et al., 2011). In this study, the content of JA and the levels of expression of the JA biosynthetic-related genes (*LOX2*, *AOS*, *OPR3*) were increased in *GhPLP2*-overexpressed plants and decreased when *GhPLP2* was knocked down (Figures 8C–F). As expected, the JA-responsive marker gene *PDF1.2* exhibited a similar trend, which is upregulated upon infection with *V. dahliae* and positively related to the content of JA and the level of expression of *GhPLP2* in plants. Therefore, *GhPLP2* may be involved in the biosynthesis of JA in Arabidopsis and cotton, which contributed to the plant defenses against *V. dahliae.*

JA is known to take part in various physiological processes including salinity, drought, and temperature (low/high) stress (Ghorbel et al., 2021). The application of exogenous JA not only increased the total carbohydrate, soluble sugar, free amino acid, total proline, and protein contents, but also the activities of catalase (CAT), POD, and SOD in maize plants (Zea mays) (Ali and Baek, 2020). In pearl millet, the exogenous application JA weakens drought stress effects on pearl millet plantlets by increasing the fresh weight of seedlings, root length and shoot length (Awan et al., 2021). In this study, the phenotype of drought sensitivity has no significant difference between *GhPLP2*-overexpressed and WT Arabidopsis plants (Supplementary Figure 10). It seems that *GhPLP2* altered JA level is not related to the plant drought response. Whether it plays a role in other abiotic stresses sharing a similar pathway with PEG 6000 treatment needs to be further determined.

Moreover, the alteration in JA-mediated pathways can also affect the vegetative and reproductive growth of plants (Qi et al., 2016; Ghorbel et al., 2021). The Arabidopsis mutant *defective in anther dehiscence1* (*dad1*) displays the reduced accumulation of JA and defects in anther dehiscence, pollen maturation, and flower opening. The exogenous application of JA or linolenic acid could rescue the defects (Ishiguro et al., 2001). In rice (*Oryza sativa*), a plastid-targeted lipase EXTRA GLUME 1 (EG1) participates in the biosynthesis of JA during the development of rice spikelets. The *eg1* mutant exhibits altered spikelet morphology with changes in floral organ identity and number, as well as defective floral meristem determinacy, resulting in a reduction in reproductive success and the yield of grains (Cai et al., 2014). In this study, *GhPLP2*-silenced cotton plants and *GhPLP2*-overexpressed Arabidopsis plants have altered JA content under mock treatment but no changes in the growing phenotype (Supplementary Figures 11A,B). This suggests that an alteration in the JA pathway through the modification of *GhPLP2* does not affect the normal growth of plants. Additionally, the overexpression of *GhPLP2* in Arabidopsis does not affect the weight of the grains (Supplementary Figure 11C). Whether the change in the JA-mediated pathway related to *GhPLP2* affects fertility and reproductive output in cotton plants is yet to be determined.

Fatty acids are not only major membrane components of the cell, but they also directly or indirectly participate in a series of immune responses in plants (Walley et al., 2013; Lim et al., 2017; He and Ding, 2020). In fact, C16:0 and 18:0 FAs serve as precursors for the synthesis of cutin and wax polymers, which provide a physical barrier to defend against insects and pathogens (Raffaele et al., 2009; Yu et al., 2013). Moreover, C18:0 fatty acid can be converted to unsaturated fatty acids including oleic acid (18:1), linoleic acid (18:2), and α-linolenic acid (18:3) after a series of desaturation reactions (Kachroo and Kachroo, 2009). Previous studies confirmed that linoleic acid (18:2) and α-linolenic acid (18:3) are precursors for JA biosynthesis (Hung and Kao, 1998; Li et al., 2003; Banilas et al., 2007). The Arabidopsis defective in anther dehiscence1 (DAD1) protein is a chloroplastic phospholipase A1 that catalyzes the initial step of JA biosynthesis by the release of JA precursor α-linolenic acid (Ishiguro et al., 2001). In cotton plants, cytochrome P450 CYP82D (SSN) competes for C18 fatty acids substrates with LOXs. The inhibition of SSN leads to free fatty acids that enter the metabolism of LOXs to synthesize JA and enhance the levels of JA and disease resistance to *V. dahliae* (Sun et al., 2014). The long non-coding RNA (lncRNA) *GhlncLOX3* positively regulates the resistance to *V. dahliae* through the modulation of the expression of *GhLOX3* implicated in α-linolenic acid (18:3) metabolism associated with JA biosynthesis (Wang et al., 2021). In this study, the contents of palmitic acid (16:0) and stearic acid (18:0) significantly increased after inoculation with *V. dahliae* in cotton and increased slightly in Arabidopsis plants. However, the difference in the contents between *GhPLP2*-silenced and control cotton plants was not significant. It is not clear whether *GhPLP2* enhances the resistance of cotton against *V. dahliae* via the regulation of accumulation of palmitic acid (16:0) and stearic acid (18:0). However, the contents of linoleic acid (18:2) and α-linolenic acid (18:3) in both Arabidopsis and cotton plants were not only closely related to the expression of *GhPLP2* under mock treatment but were also increased following infection with *V. dahliae* (Figures 8A,B). These findings suggest that *GhPLP2* mediates the production of linoleic acid (18:2) and α-linolenic acid (18:3), which are involved in plant defense against *V. dahliae*. Free C18:2 and C18:3 was reduced to stable hydroxy FA in the cytosol or plastids and then converted to oxylipins which including JAs (Feussner and Wasternack, 2002; La Camera et al., 2009). Therefore, we hypothesize that the production of linoleic acid and α-linolenic acid by *GhPLP2* may promote the biosynthesis and signaling of JA.

HR is one of the defense mechanisms that confers the plant with the ability to inhibit pathogen infection and further induce a rapid defense response (Kombrink and Schmelzer, 2001; Zhang Y. et al., 2017). Interestingly, several oxylipins that are derived from fatty acids in the 9- and 13-LOX pathways were found to be sufficient to initiate HR in different pathosystems (García-Marcos et al., 2013; Sun et al., 2014). Increasing amounts of evidence showed that the PLPs play an essential role in the execution of cell death in HR in plants. In Arabidopsis, *AtPLP2* contributes to the resistance response to *Cucumber mosaic virus* (*CMV*) upon HR-dependent process (La Camera et al., 2009). *CaPLP1* regulates the production of phospholipid-derived molecules, which leads to the HR-like cell death during the incompatible interaction of pepper plants with avirulent *Xcv* (Kim et al., 2014). The elicitor protein PevD1 from *V. dahliae* can induce typical HR-like necrosis in tobacco and trigger innate immunity in cotton plants. *GhPLP2* silencing has reduced the degree of HR and expression of the HR marker gene *GhHIN1* and *GhHSR203* during the induction of *V. dahliae* elicitor PevD1 in cotton leaves (Figure 6). This suggests that *GhPLP2* is required for resistance (R) gene-mediated disease resistance in cotton plants. However, further research is merited to reveal the relationship between GhPLP2 and PevD1-triggered HR during the interaction between cotton and *V. dahliae*.

**FIGURE 9:**
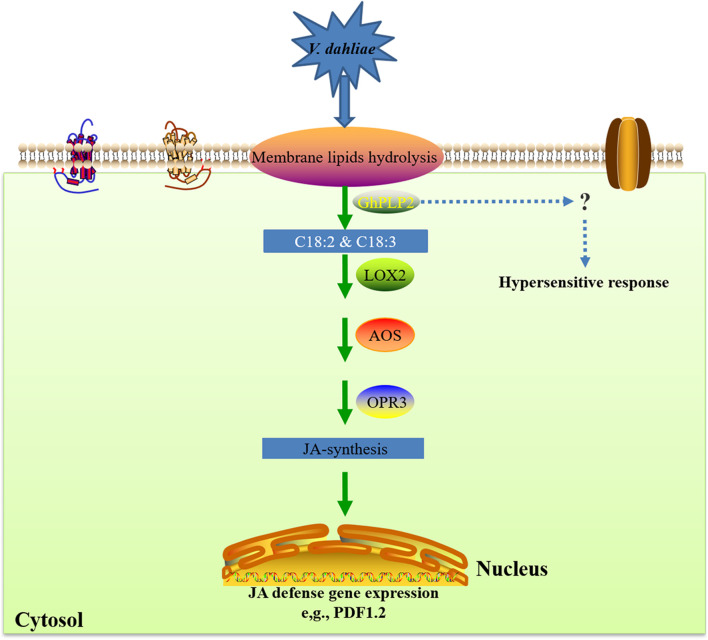
A proposed model for the involvement of *GhPLP2* in plant resistance against *Verticillium dahliae*. LOX, lipoxygenase; AOS, allene oxide synthase; OPR3, *cis*-(+)-12-oxo-phytodienoic acid reductase 3.

Taken together, we propose a potential model of the involvement of *GhPLP2* in the resistance of plants against *V. dahliae* (Figure 9). *GhPLP2* contributes to the build-up of free fatty acids pools, including linoleic acid and α-linolenic acid. The accumulation of linoleic acid and α-linolenic acid may directly or indirectly lead to the activation of the JA-synthesis and -signaling pathway in plants; *GhPLP2* is also involved in PevD1-triggered HR. Both in concert enhance the resistance of cotton plants to disease caused by infection with *V. dahliae*.

## Conclusion

Our study provides evidence that the patatin-like protein GhPLP2 with LAH activity plays a positive role in the enhancement of plant disease resistance. The silencing of *GhPLP2* in cotton plants attenuated resistance against *V. dahliae*, and the overexpression of *GhPLP2* in Arabidopsis enhanced resistance against infection with *V. dahliae*. The resistance mechanism of *GhPLP2* may be owing to its release of the synthetic precursors to jasmonic acid, including linoleic acid and α-linolenic acid, via its LAH activity, followed by the accumulation of JA and activation of the JA signaling pathway. In addition, *GhPLP2* is involved in triggering a hypersensitive response for disease resistance by a *V. dahliae* elicitor. These results stressed some insights for the mechanism of *GhPLP2* in the response of plant defenses and highlighted the potential application of *GhPLP2* in genetic engineering to increase the disease resistance of cotton germplasm.

## Data Availability Statement

The datasets presented in this study can be found in online repositories. The names of the repository/repositories and accession number(s) can be found in the article/Supplementary Material.

## Author Contributions

YH, FL, and YZ conceived and designed the study. YZ conducted most of the experiments and wrote the manuscript. XH, LG, and ZG provided technical assistance to YZ. YP and PW provided analysis tools. XG contributed reagents and materials. All authors reviewed and approved the final manuscript.

## Conflict of Interest

The authors declare that the research was conducted in the absence of any commercial or financial relationships that could be construed as a potential conflict of interest.

## Publisher’s Note

All claims expressed in this article are solely those of the authors and do not necessarily represent those of their affiliated organizations, or those of the publisher, the editors and the reviewers. Any product that may be evaluated in this article, or claim that may be made by its manufacturer, is not guaranteed or endorsed by the publisher.

## Footnotes

1
https://cottonfgd.org/

2
http://web.expasy.org/protparam/

3
http://www.ncbi.nlm.nih.gov

4
https://www.ebi.ac.uk/Tools/msa/clustalo/

5
https://embnet.vital-it.ch/software/BOX_form.html

6
https://meme-suite.org/meme/tools/meme
