# Supplementary material for: GhPLP2 Positively Regulates Cotton Resistance to Verticillium Wilt by Modulating Fatty Acid Accumulation and Jasmonic Acid Signaling Pathway
Source: Front Plant Sci. 2021 Nov 2;12:749630. doi: 10.3389/fpls.2021.749630 (PMC8593000; doi:10.3389/fpls.2021.749630)
Supplement: Supplementary file 1 [file Data_Sheet_1.ZIP › Electronic Supplementary Material/Supplementary Figure 10.pdf]

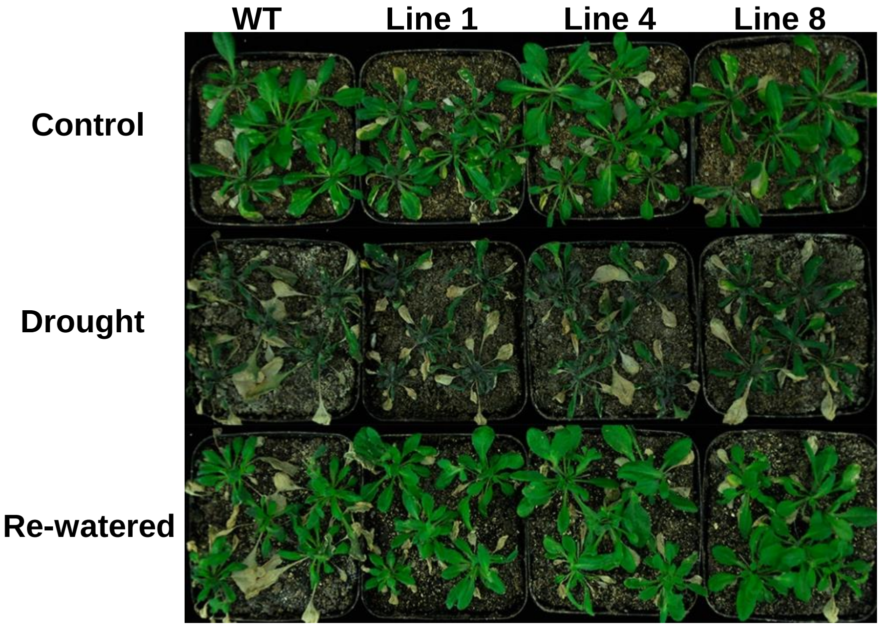

Supplementary Figure 10. The phenotype of drought sensitivity of *GhPLP2*-overexpressed and WT Arabidopsis Plants. Phenotype of drought sensitivity in WT and *GhPLP2*-overexpressed Arabidopsis plants grown in a growth chamber without watering for 16 d. Take a photo after two days with watering. The control plants were cultured under normal conditions.
