# Supplementary material for: GhPLP2 Positively Regulates Cotton Resistance to Verticillium Wilt by Modulating Fatty Acid Accumulation and Jasmonic Acid Signaling Pathway
Source: Front Plant Sci. 2021 Nov 2;12:749630. doi: 10.3389/fpls.2021.749630 (PMC8593000; doi:10.3389/fpls.2021.749630)
Supplement: Supplementary file 1 [file Data_Sheet_1.ZIP › Electronic Supplementary Material/Supplementary Figure 11.pdf]

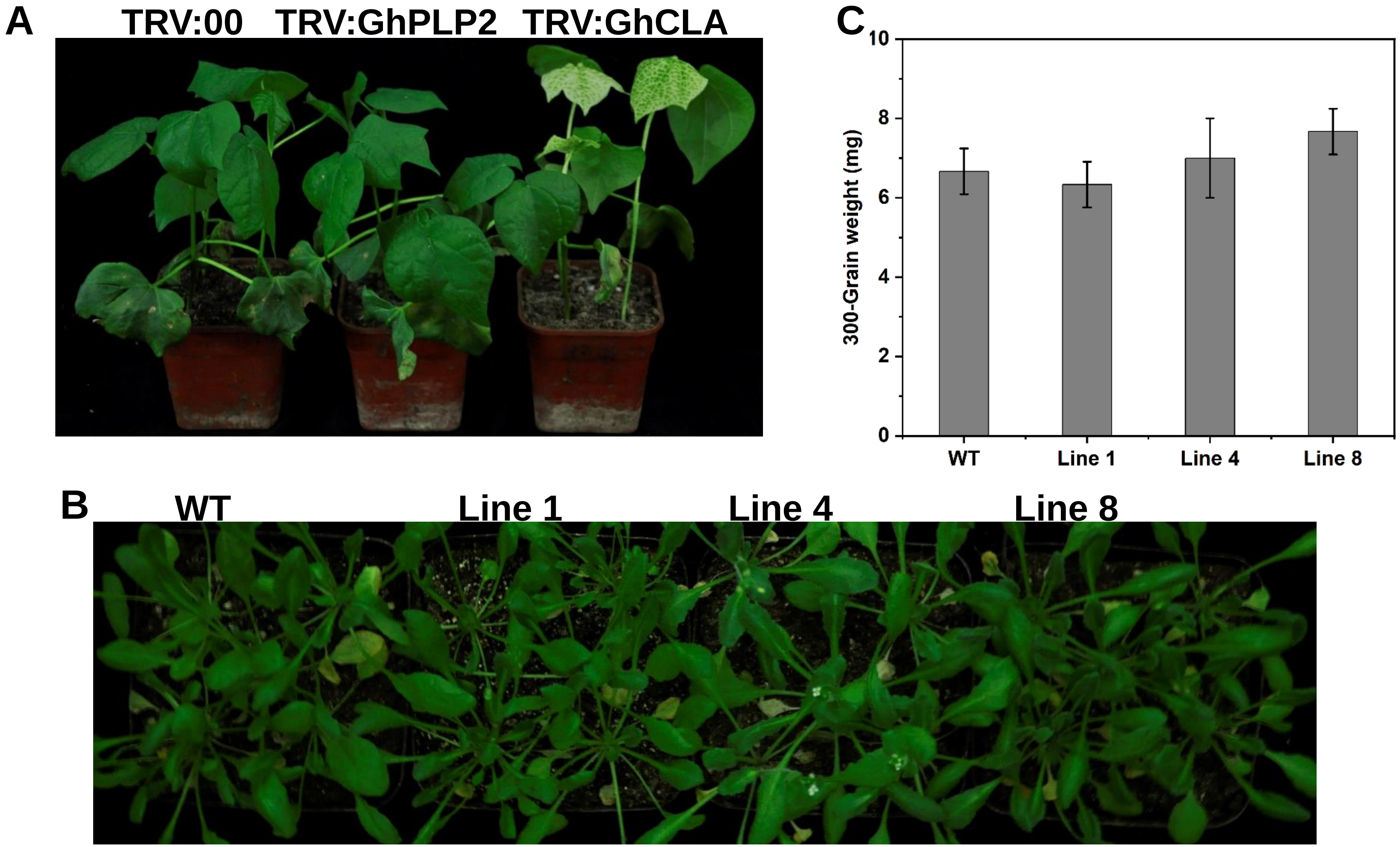

Supplementary Figure 11. The growth phenotype of Arabidopsis and cotton plants. (**A**) The growth phenotype of cotton plants after two weeks of VIGS. (**B**) The growth phenotype of four-week-old *GhPLP2*-transgenic and WT Arabidopsis plants. (**C**) Quantitation of 300-grain weight of WT and *GhPLP2*-overexpressed Arabidopsis seeds.
