# Supplementary material for: GhPLP2 Positively Regulates Cotton Resistance to Verticillium Wilt by Modulating Fatty Acid Accumulation and Jasmonic Acid Signaling Pathway
Source: Front Plant Sci. 2021 Nov 2;12:749630. doi: 10.3389/fpls.2021.749630 (PMC8593000; doi:10.3389/fpls.2021.749630)
Supplement: Supplementary file 1 [file Data_Sheet_1.ZIP › Electronic Supplementary Material/Supplementary Figure 4.pdf]

A

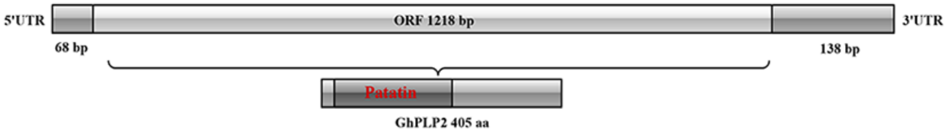

B

```
1     ATGGAAAAAAGTACTGGAAACCCTAATCACCCTCCATGTCATGGAGATCTTATCACCGTT
       M  E  K  S  T  G  N  P  N  H  P  P  C  H  G  D  L  I  T  V   20
61    CTCAGCATTGATGGAGGTGGAATTAGGGGAATCATTCCAGGAATAATTCTCAGCTTTTTA
       L  S  I  D  G  G  G  I  R  G  I  I  P  G  I  I  L  S  F  L   40
121   GAATCTGAGTTACAAAAACTGGACAGTGAAGAAGCAAGAATTGCTGATTATTTTGATGTG
       E  S  E  L  Q  K  L  D  S  E  E  A  R  I  A  D  Y  F  D  V   60
181   ATCGCCGGAACTAGCACCGGCGGTCTCCTGACCACCATGTTAACATCTCCCAACGAGAAA
       I  A  G  T  S  T  G  G  L  L  T  T  M  L  T  S  P  N  E  K   80
241   AACCGACCCCTATTTGCTGCCAAAGACATCAAGGCCTTCTACTTCGAGCATGGCCCTAAA
       N  R  P  L  F  A  A  K  D  I  K  A  F  Y  F  E  H  G  P  K   100
301   ATATTCCCTCAAAGAAGGTTTCCATTTTCTGGTGTTACAAAAATTATTCAAAGTGTGATG
       I  F  P  Q  R  R  F  P  F  S  G  V  T  K  I  I  Q  S  V  M   120
361   GGACCAAAATATGATGGCAAGTATCTGCATGGTGTTCTTAAGGAAAGATTAGGAAACACA
       G  P  K  Y  D  G  K  Y  L  H  G  V  L  K  E  R  L  G  N  T   140
421   AGGCTGAACCAGACACTGACTAACGTTGTAATTCCAACTTTCGATATCAAGAAACTTCAG
       R  L  N  Q  T  L  T  N  V  V  I  P  T  F  D  I  K  K  L  Q   160
481   CCCATTATCTTTTCTAGCTATGAGGTGAAGAAATCTGCAGGCCTTAATGCCTTAATGTCA
       P  I  I  F  S  S  Y  E  V  K  K  S  A  G  L  N  A  L  M  S   180
541   GACATTTGCATTGGAACTTCAGCCGCTCCAACTTATCTGCCGGCCCATTATTTTGAAACC
       D  I  C  I  G  T  S  A  A  P  T  Y  L  P  A  H  Y  F  E  T   200
601   GAGGACACTGATGGAAAGGTTAAAAAGTTCAATCTTATCGATGGTGGGGTGGCTGCTAAT
       E  D  T  D  G  K  V  K  K  F  N  L  I  D  G  G  V  A  A  N   220
661   AATCCGGCTCTAATTGCCATGGGTGAAGTGACAAAACAGATTCACAAAGGCCATTCAGAT
       N  P  A  L  I  A  M  G  E  V  T  K  Q  I  H  K  G  H  S  D   240
721   TTTTCTTGCATTAAAGCAGCAAATCATTACAATAGATTCTTGGTGCTATCCGTTGGAACA
       F  S  C  I  K  A  A  N  H  Y  N  R  F  L  V  L  S  V  G  T   260
781   GGCTCAGAAAAACTAGCAGAGAAATACACTGCAAAGCAAGCGGCTCAATGGGGCGTACTT
       G  S  E  K  L  A  E  K  Y  T  A  K  Q  A  A  Q  W  G  V  L   280
841   GGTTGGTTAACCTCTGGCAATTCAACCCCATTAATCAATGCCTTTTCCGAAGGCAGTGCT
       G  W  L  T  S  G  N  S  T  P  L  I  N  A  F  S  E  G  S  A   300
901   GATATGGTTGATTTCCACATTTCCGTGATTTTCAAATCTTTAAACTCCGAGCCGAATTAT
       D  M  V  D  F  H  I  S  V  I  F  K  S  L  N  S  E  P  N  Y   320
961   CTTCGCATCCAGGATGATAATCTGAAAGGGGAGGTTTCCTCTGTGGATGTTTCAACGGAG
       L  R  I  Q  D  D  N  L  K  G  E  V  S  S  V  D  V  S  T  E   340
1021  GAAAATATGAAAATTTTAGCTAAAGTAGCAGAAAACTTACTGAATAAACCAGTTTCAAGG
       E  N  M  K  I  L  A  K  V  A  E  N  L  L  N  K  P  V  S  R   360
1081  GTGAATTTCGAGACGGGAAATTATGAACCTTCAGGAGACTTAGAAACCAATGCTCAAGCT
       V  N  F  E  T  G  N  Y  E  P  S  G  D  L  E  T  N  A  Q  A   380
1141  CTCATAAGGTACGCGAAGCTGCTGTCTGAGGAAAAACGCCGACGTCTAAAGACATCGTTG
       L  I  R  Y  A  K  L  L  S  E  E  K  R  R  R  L  K  T  S  L   400
1201  CACAAGCTTGGCCCATAA
       H  K  L  G  P  *                                             405
```

Supplementary Figure 4. (**A**) Diagrams of the nuclotide and deduced amino acid sequences of GhPLP2. (**B**) Nuclotide sequence and deduced amino acid sequence of GhPLP2. The red area is the patatin domain.
