# Supplementary material for: GhPLP2 Positively Regulates Cotton Resistance to Verticillium Wilt by Modulating Fatty Acid Accumulation and Jasmonic Acid Signaling Pathway
Source: Front Plant Sci. 2021 Nov 2;12:749630. doi: 10.3389/fpls.2021.749630 (PMC8593000; doi:10.3389/fpls.2021.749630)
Supplement: Supplementary file 1 [file Data_Sheet_1.ZIP › Electronic Supplementary Material/Supplementary Figure 5.pdf]

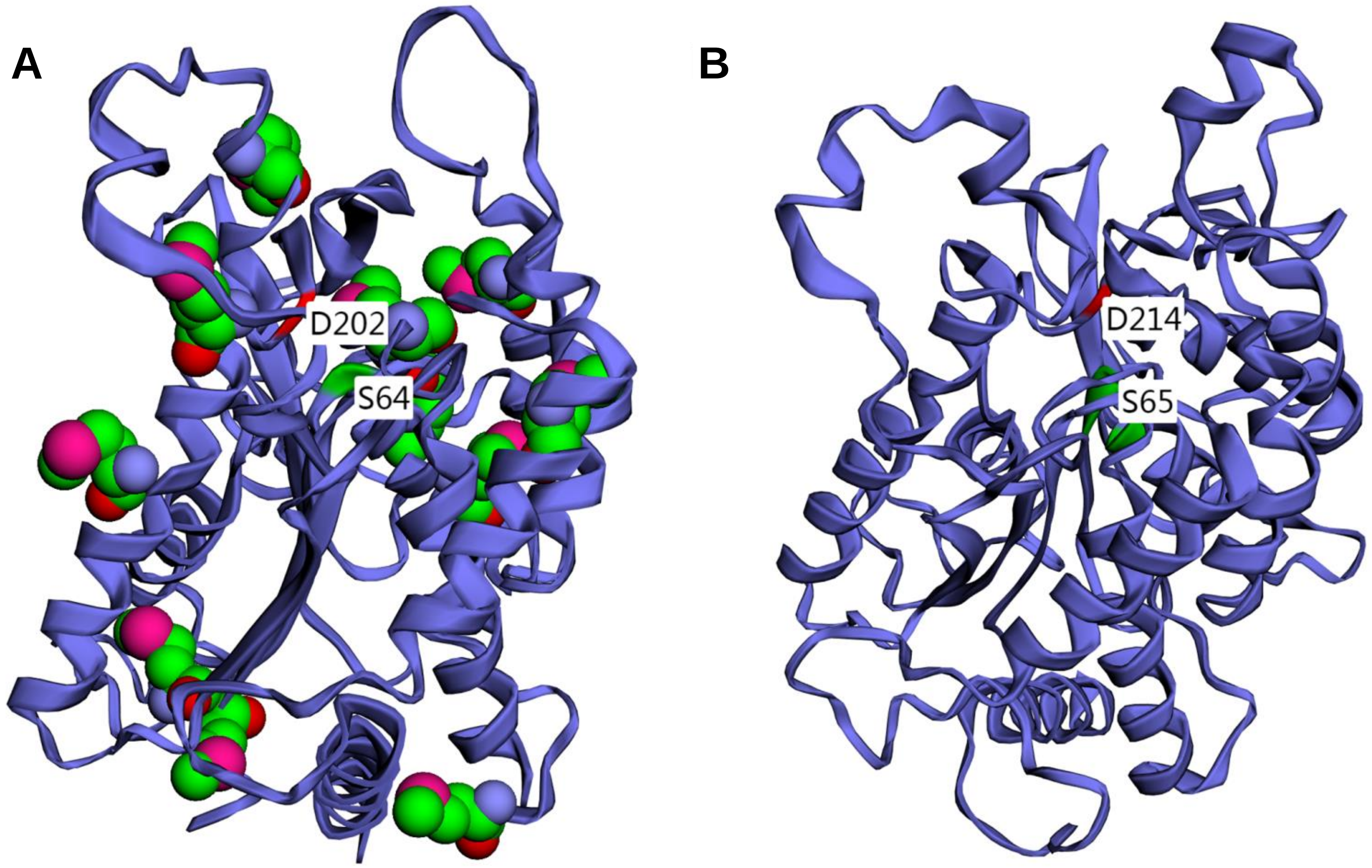


Supplementary Figure 5. Structure predictions of GhPLP2 and SeMet Patatin (40.22% similarity). (**A**) The crystal structure of SeMet Patatin (PDB number:1oxw.1.B). Amino acids Ser (S64) and Asp (D202) are highlighted. (**B**) Modeling of GhPLP2 protein. Amino acids Ser (S65) and Asp (D214) are highlighted. The active sites composed of Ser-Asp catalytic dyad responsible for its LAH activity.
