# Supplementary material for: GhPLP2 Positively Regulates Cotton Resistance to Verticillium Wilt by Modulating Fatty Acid Accumulation and Jasmonic Acid Signaling Pathway
Source: Front Plant Sci. 2021 Nov 2;12:749630. doi: 10.3389/fpls.2021.749630 (PMC8593000; doi:10.3389/fpls.2021.749630)
Supplement: Supplementary file 1 [file Data_Sheet_1.ZIP › Electronic Supplementary Material/Supplementary Figure 6.pdf]

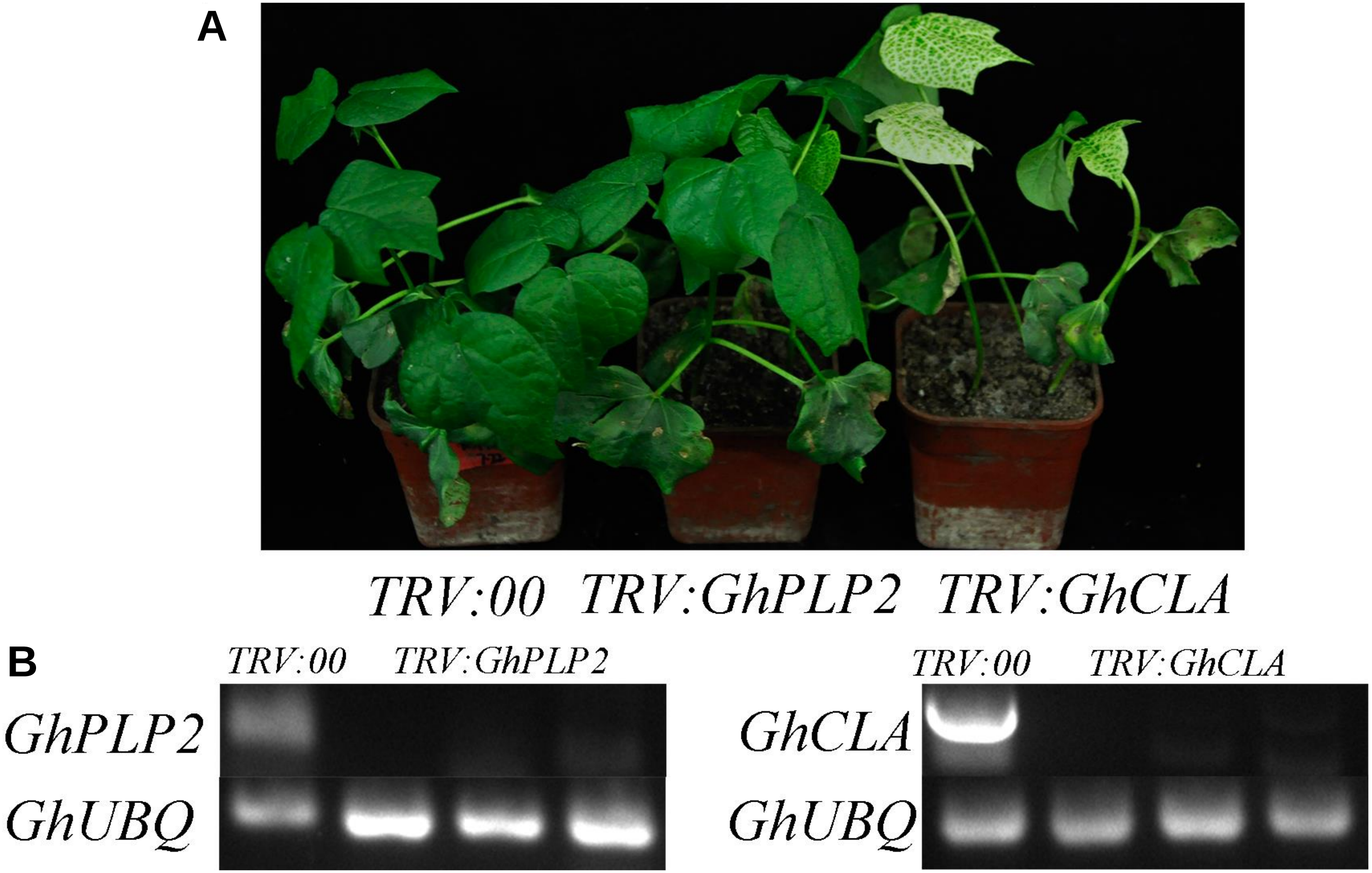

Supplementary Figure 6. Gene silencing in cotton plants by VIGS. (**A**) The phenotypes of *TRV:00*, *TRV:GhPLP2*, *TRV:GhCLA* cotton plants after two weeks of VIGS. (**B**) The expression of *GhPLP2* and *GhCLA* in the control and silenced cotton were analyzed by semi-quantitative qPCR. *GhUBQ7* was used as a reference gene.
