# Supplementary material for: GhPLP2 Positively Regulates Cotton Resistance to Verticillium Wilt by Modulating Fatty Acid Accumulation and Jasmonic Acid Signaling Pathway
Source: Front Plant Sci. 2021 Nov 2;12:749630. doi: 10.3389/fpls.2021.749630 (PMC8593000; doi:10.3389/fpls.2021.749630)
Supplement: Supplementary file 1 [file Data_Sheet_1.ZIP › Electronic Supplementary Material/Supplementary Figure 7.pdf]

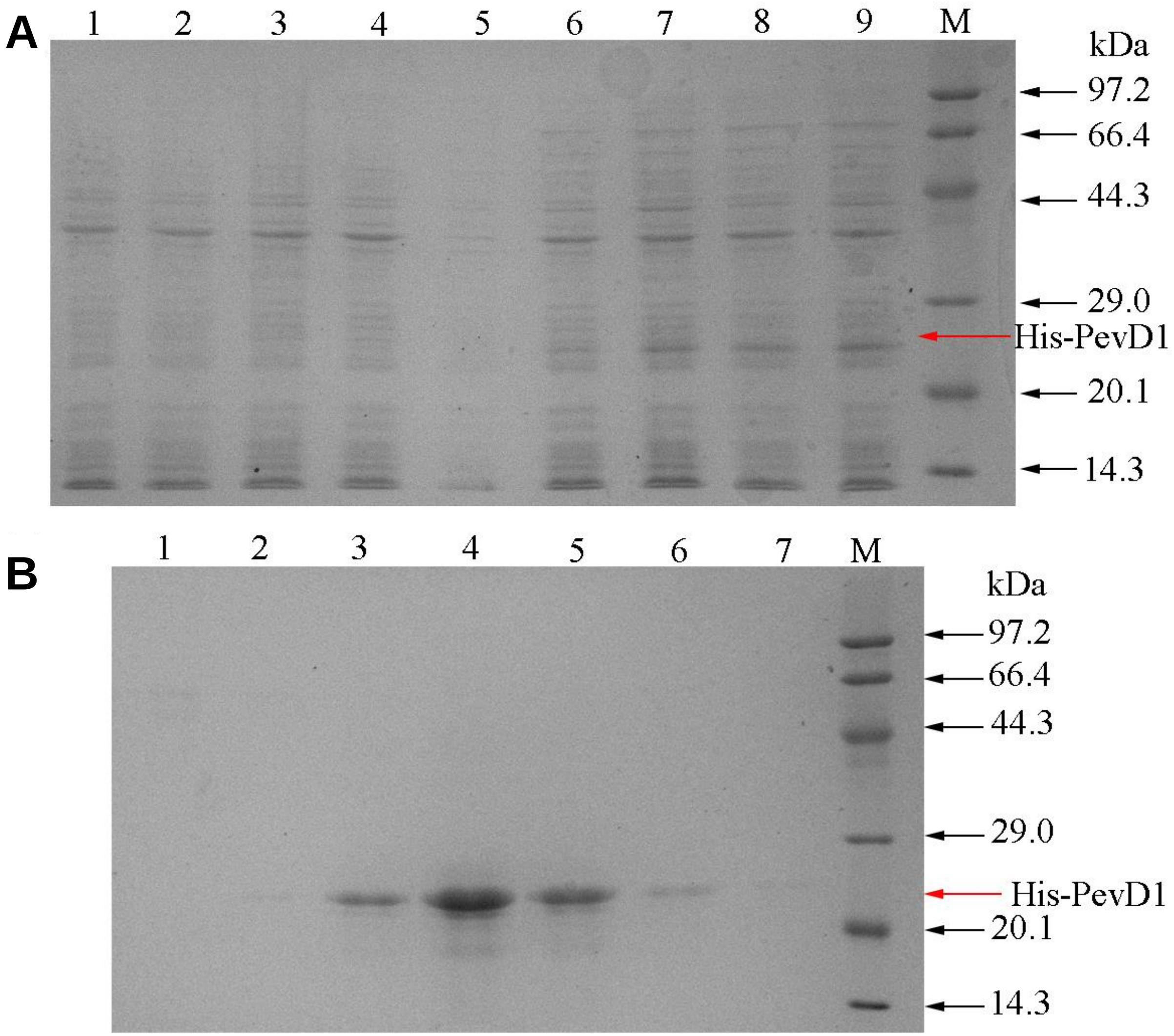

Supplementary Figure 7. Purification of recombinant PevD1 protein. (**A**) Proteins from induced bacteria were analyzed by SDS-PAGE. 1-4, pET-28a empty vector was induced at 5 h, 10 h, 15 h, 20 h. 5-9, pET-28a-PevD1 was induced at 0, 5 h, 10 h, 15 h, 20 h. 0.1 mM IPTG, 22 °C, 200 rpm. M molecular mass markers (kDa). (**B**) Purification of recombinant PevD1. M: molecular mass of protein marker. 1-7, different elution proteins.
