# Supplementary material for: GhPLP2 Positively Regulates Cotton Resistance to Verticillium Wilt by Modulating Fatty Acid Accumulation and Jasmonic Acid Signaling Pathway
Source: Front Plant Sci. 2021 Nov 2;12:749630. doi: 10.3389/fpls.2021.749630 (PMC8593000; doi:10.3389/fpls.2021.749630)
Supplement: Supplementary file 1 [file Data_Sheet_1.ZIP › Electronic Supplementary Material/Supplementary Figure 8.pdf]

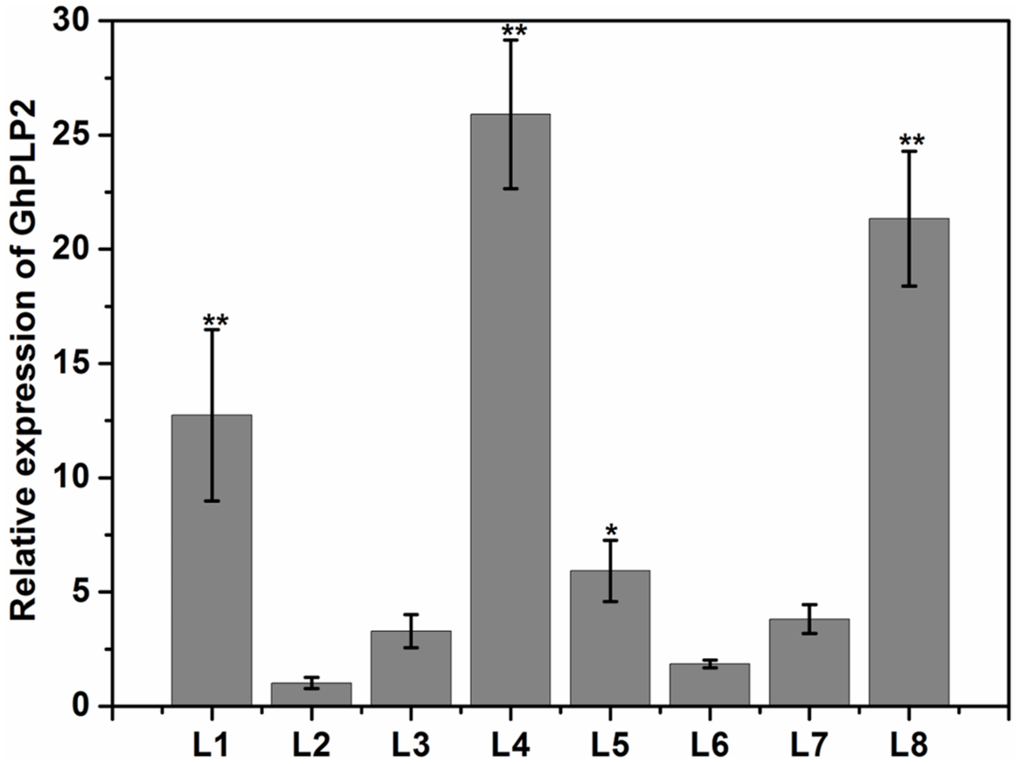

Supplementary Figure 8. Transcript levels of *GhPLP2* was analysised by qRT-PCR in transgenic *Arabidopsis* lines (whole plants) relative to the line with the lowest expression (L2). *AtEF1α* (AT5G60390) was employed as the internal standard. Data were collected from three independent biological samples . Error bars represent standard error. Asterisks indicate a significant difference (*P < 0.05, **P <0.01, Student's *t*-test).
