# Supplementary material for: GhPLP2 Positively Regulates Cotton Resistance to Verticillium Wilt by Modulating Fatty Acid Accumulation and Jasmonic Acid Signaling Pathway
Source: Front Plant Sci. 2021 Nov 2;12:749630. doi: 10.3389/fpls.2021.749630 (PMC8593000; doi:10.3389/fpls.2021.749630)
Supplement: Supplementary file 1 [file Data_Sheet_1.ZIP › Electronic Supplementary Material/Supplementary Figure 9.pdf]

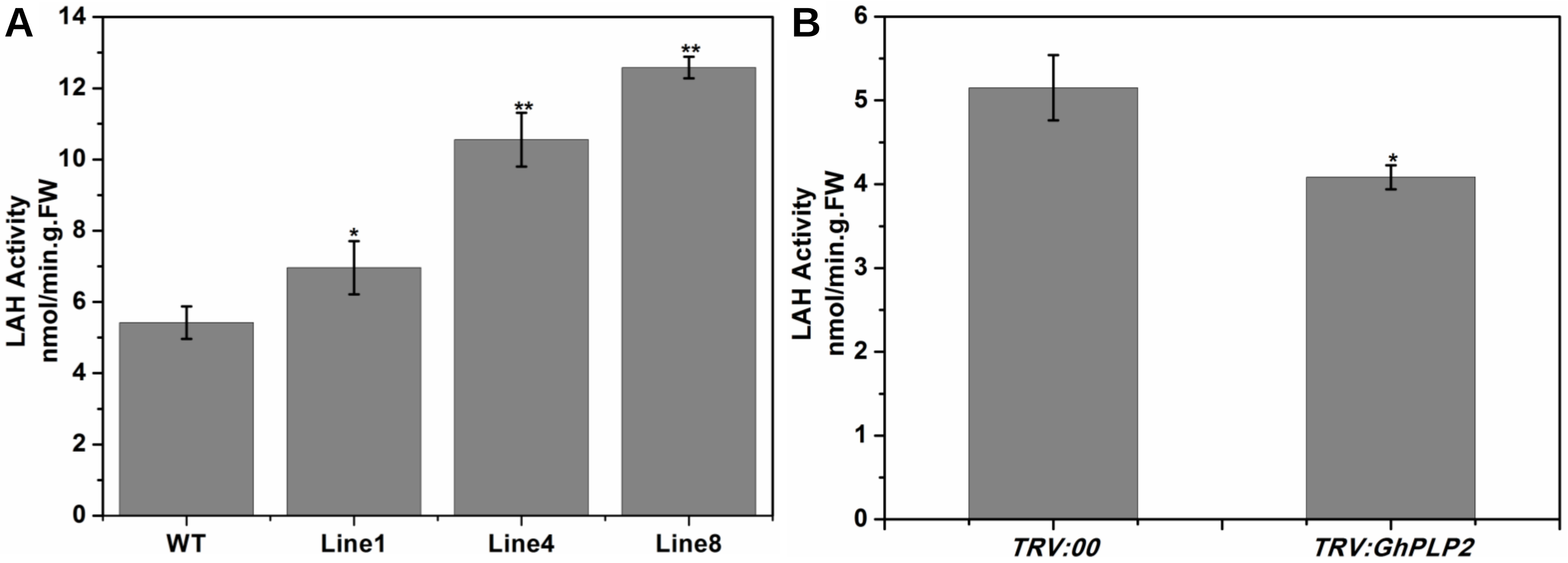

Supplementary Figure 9. The endogenous LAH activity of crude proteins from different genotypes plants were detected using p-nitrophenyl palmitate (p-NPP). (**A**) The endogenous LAH activity of crude proteins from *GhPLP2*-transgenic Arabidopsis. (**B**) The endogenous LAH activity of crude proteins from *TRV:00* and *TRV:GhPLP2* cotton plants. Data were collected from three independent biological replicates. Error bars represent standard error. Asterisks indicate a significant difference (**P* < 0.05, ***P* <0.01, Student's *t*-test).
